# A framework for establishing shared, task-oriented understanding in hybrid open multi-agent systems

**DOI:** 10.3389/frai.2025.1440582

**Published:** 2025-04-16

**Authors:** Nikolaos Kondylidis, Ilaria Tiddi, Annette ten Teije

**Affiliations:** Computer Science, Vrije Universiteit Amsterdam, Amsterdam, Netherlands

**Keywords:** human-agent communication, task-oriented understanding establishment, hybrid open multi-agent systems, shared understanding, human-agent collaboration

## Abstract

In Open Multi-Agent Systems (OMAS), the open nature of such systems precludes that all communication protocols are hardwired in advance. It is therefore essential that agents can incrementally learn to understand each other. Ideally, this is done with a minimal number of a priori assumptions, in order not to compromise the open nature of the system. This challenge becomes even harder for hybrid (human-artificial agent) populations. In such a hybrid setting, the challenge of learning to communicate is exacerbated by the requirement to do this in a minimal number of interactions with the humans involved. The difficulty arises from the conflict between making a minimal number of assumptions while also minimizing the number of interactions required. This study provides a fine-grained analysis of the process of establishing a shared task-oriented understanding for OMAS, with a particular focus on hybrid populations, i.e., containing both human and artificial agents. We present a framework that describes this process of reaching a shared task-oriented understanding. Our framework defines components that reflect decisions the agent designer needs to make, and we show how these components are affected when the agent population includes humans, i.e., when moving to a hybrid setting. The contribution of this paper is not to define yet another method for agents that learn to communicate. Instead, our goal is to provide a framework to assist researchers in designing agents that need to interact with humans in unforeseen scenarios. We validate our framework by showing that it provides a uniform way to analyze a diverse set of existing approaches from the literature for establishing shared understanding between agents. Our analysis reveals limitations of these existing approaches if they were to be applied in hybrid populations, and suggests how these can be resolved.

## 1 Introduction

Computers and humans are progressively engaged in collaborative relationships, either for the purpose of negotiation or task delegation (Akata et al., 2020; Billhardt et al., 2014). Smooth interaction between human and artificial agents depends on their ability to “exchange knowledge and information across the boundary between computational space and the human space” (Billhardt et al., 2014). In such envisioned human-agent collaborations, not all interaction scenarios can be foreseen, and there will be cases where the interacting agents will need to extend their shared understanding in order to achieve their goals.

In any Multi-Agent System (MAS), agents need to transmit information or knowledge to each other, in order to cooperate, plan, and in general to decide how to behave toward achieving their goals. In order to keep such OMAS as open as possible, the set of assumptions that must be shared by all participating agents should be kept to a minimum. This becomes very challenging in Open MAS (OMAS), i.e., where the population of agents is dynamic, and it cannot be assumed that all needed concepts can be communicated across agents over existing shared languages. New signals, e.g., symbols, words, etc. can be communicated, but their interpretation needs to be aligned on the fly through interaction, a process we describe as establishing a shared understanding. This becomes additionally difficult when the agent population is hybrid, i.e., includes humans and artificial agents. First, humans do not operate using formal semantics, making it hard to define and validate communication languages or interpretations. Second, in hybrid settings, the number of interactions with human agents is much more strictly bound than between artificial agents.

Many methods for establishing shared understanding in OMAS have been published in the literature (Atencia and Schorlemmer, 2012; Rovatsos et al., 2003; Steels and Loetzsch, 2012; Mohan and Laird, 2014; Kondylidis et al., 2023; Euzenat, 2014; Laera et al., 2007; Anslow and Rovatsos, 2015), to name a few. This paper does not provide another method for doing so. Instead, it aims to describe the process of establishing shared understanding itself. Other studies represent formally human communication, either based on speech act theory and theory of action (Mann, 1988), or under the assumption that all communication can be represented with commitments (Walton and Krabbe, 1995). Other studies propose formal frameworks that model human or computational agent communication (Prakken, 2009; Atkinson et al., 2012; Hulstijn et al., 2005; Dignum et al., 2001). These formal definitions allow us to represent agent interactions, calculate conversation outcomes, or to construct formal communication languages for OMAS, enabling computational agents to negotiate, inquire, and persuade each other. Some of these frameworks have components similar to ours, as we see in Section 2. Nevertheless, we argue that these frameworks operate using shared semantics, i.e., languages that the agents already interpret in the same way, in contrast to the framework we put forward in this paper. Specifically, as also described in Section 2.4.4, they enable the agents to negotiate over propositions that are defined over commonly understood semantics. For example, they can argue if “agent A will take out the trash,” while the participants already agree on “who is agent A,” “what is the trash,” and what it means to “take something out.” In this work, we aim to describe the process of establishing shared understanding in OMAS systematically, while pointing out what requires further attention in the subcase of hybrid populations.

In this study, we analyze the process in which two agents learn to understand each other, i.e., establish shared understanding. In our framework, the agents need not understand everything in exactly the same way. Instead, they only have to understand a set of concepts sufficiently similarly to fulfill the needs of a particular task they collectively want to perform, such as answering a query or performing an action. When this is achieved, we say that the agents have established a *shared task-oriented understanding*. Establishing such a shared task-oriented understanding is an iterative process that extends some prior shared understanding toward successfully communicating a broader set of concepts required for the task. Prior shared understanding can for example be the ability to refer to world's objects or to assume the intentions of other agents. Our work is based on the idea that interactions within hybrid populations can be studied as a specific case of OMAS, i.e., hybrid OMAS, as also suggested by Hulstijn et al. (2005). A framework for such hybrid OMAS should explain how two agents can learn to understand each other through interaction, and to point out which parts require special attention in case of human participation. To this end, this paper answers the following three research questions:

1. *Can we design a framework to represent the process of establishing shared task-oriented understanding in hybrid OMAS?*
2. *Can this framework help us identify whether two agents indeed successfully establish shared understanding?*
3. *Does this framework help us to identify limitations of existing studies if they were to be applied to hybrid populations?*

The framework that we propose in this paper consists of three groups of components. Each component comes with a question that describes the role of the component in the process, in order to assist agent policy designers. The first group, named “Preliminaries,” has 4 components (P1–P4) and encapsulates what needs to be defined prior to the interaction. The second group, called “Interaction,” has 6 components (I1–I6) and describes the agent interaction and its learning outcomes. The last category has the 4 “Agent Policy” components (A1–A4) and describes the agent's behavior regarding the interactions and toward achieving shared goal oriented understanding. Finally, we point out which restrictions these components must adhere to when the agent population includes humans (Kondylidis et al., 2023). Our proposed framework is intended to be used as a resource during the process of designing agents that need to interact with humans to establish shared understanding in unforeseen scenarios.

The utility of the framework is evaluated on its ability to analyze and describe a broad spectrum of existing (hybrid) OMAS studies of establishing shared agent understanding. These studies come from a broad set of domains, ranging from agent-based ontology alignment and reward-driven OMAS to language games and human-computer concept teaching based on interaction, ensuring a broad validity of the framework we propose. Our framework successfully analyzes the process of establishing shared understanding for each case and further helps us understand similarities and differences among this range of studies. Additionally, for some of the studies, it identifies the reasons hindering their application in hybrid populations and provides possible solutions of how these can be overcome.

In the next section, we present related studies and position our proposed framework accordingly. Furthermore, in Section 3, we present our definitions of individual and shared task-oriented understanding, and shared understanding. We then define our framework, its components, and how some of them are affected in case of hybrid populations. In Section 4, we apply our framework to a number of existing OMAS studies, allowing us to illustrate the utility of the proposed framework and answer our research questions. In the last section, we conclude with directions for future work.

## 2 Related work

In this section, we will review related studies and position our work according to them. The problem of information and knowledge exchange between agents has been approached by different fields. The field of ontology alignment aims to provide semantic translations between databases defined in different schemas, allowing information defined in some schema to be interpreted using concepts of another (Euzenat et al., 2007). These approaches either require complete access to both schemata or databases, or it can be too inefficient or cognitively demanding to be used in a hybrid OMAS setting (Kondylidis et al., 2023). OMAS studies must also tackle the establishment of agent interoperation on the fly, which needs to be built on top of successful agent communication. Several methods have been proposed for this purpose, Steels and Loetzsch (2012); Euzenat (2014); Laera et al. (2007); Atencia and Schorlemmer (2012); Anslow and Rovatsos (2015); Rovatsos et al. (2003) to name a few. Most of these studies propose a method that allows agents to interoperate for some specific interaction setting or task, like query answering, but do not study the process itself as we aim to do in this work. Additionally, the application range of these studies is computational systems and cannot be seamlessly applied to hybrid populations, either due to interaction or inefficiency constraints, as described in Section 3.4. On the other hand, some studies focus on structuring human or computational agent interaction and represent it formally, sometimes using a framework. These studies will be presented in Section 2.4 together with how they relate with our framework and their differences. Finally, we go over guidelines for establishing shared understanding in hybrid populations as put forward by Kondylidis et al. (2023), that are incorporated in the presented work.

### 2.1 Lewis signaling game

Modeling the scenario where two agents need to communicate in order to achieve a common goal goes at least as far back as the Lewis signaling game (Lewis, 1969). In this game, two agents, i.e., sender and receiver, exist in an environment that is in one state, from a discrete set of states. For each state, there is an action, preferred by both agents, making it a game of pure common interest. What requires communication is the fact that only the sender knows the environment state, and only the receiver can perform an action. The sender produces a (discrete) signal as an attempt to inform the receiver about the state of the environment, so that the receiver can make an informative decision when selecting an action. The Lewis signaling game is a more abstract version of our Interaction components (I1–I6), describing how two agents relate communication acts with states or actions in order to coordinate. In this work, we show how agent interaction is actually framed in the proposed shared task-oriented understanding framework, in which the interactions act as a tool but are not the end goal.

### 2.2 Language games

Language Games (Steels and Loetzsch, 2012) is a well-known stream of studies where a population of agents develop and use their own language successfully. The interacting agents, i.e., Speaker and Listener, engage in referential games, an extension of the Lewis signaling game. The Speaker may create a new word if necessary, and at the end of the interaction it reveals the correct behavior to the Listener, if the latter one deviated. Over time the agents can learn to interpret a common vocabulary of signals, i.e., words, in a similar enough manner, allowing them to successfully communicate in all provided scenarios. While the agents interact in pairs of randomly selected agents from the population, their pairwise vocabulary semantic convergence generalizes over the complete population, after enough interactions and when all agents have participated several times. An important takeaway from these studies is that the agents in practice never use the exact same word interpretations, and that this is not necessary, as long as the interpretations are similar enough in the presented context. This proves that a system of agents with heterogeneous interpretation can in practice establish a shared task-oriented understanding and communicate successfully, as long as the interpretations are similar enough, with respect to the context and to the extent that the task requires them to be. This finding allowed us to conceptualize our framework for heterogeneous agents, while also supporting the idea that a shared (task-oriented) understanding can be indirectly measured according to task performance. The authors perform experiments to show how shared task-oriented understanding can be established for the specific case, where a population of robotic agents play the referential game. As such, it is further analyzed among other studies in Section 4. In contrast, we define a general framework that formalizes this process from any setting of agents or tasks, while also the types of (shared) understanding and how they can be measured. Additionally, we point out restrictions that must take place when the agent population is hybrid.

### 2.3 Cooperation and alignment in human dialogues

How humans learn to understand each other through dialogues has also been largely studied (Pickering and Garrod, 2004). Specifically, Pickering and Garrod (2004) suggests that humans have used situation models to communicate, i.e. comprehend or produce language. Furthermore, situation models and linguistic representations of two people engaging in a dialogue align over time, as their alignment is the basis for successful dialogue (Zwaan and Radvansky, 1998). Furthermore, humans do not have to use the same situation models in order to communicate, instead they only need to establish a “common ground,” i.e., share the same beliefs and knowledge about discussed concepts. Humans in practice seem to infer an implicit common ground, which only needs expansion and further alignment when a misunderstanding occurs. Since humans do not need to use the same situation models or explicitly defined common ground, we argue that they can also establish implicit common ground with computational agents and also learn to understand them too, i.e., establish shared (task-oriented) understanding. Finally, Pickering and Garrod (2004) use the terms “coordination” and “alignment,” which we define in Section 3.2 as “shared task-oriented understanding” and “shared understanding,” respectively. The former reflects the ability of two agents to understand each other in order to accomplish a task that requires their coordination. The latter suggests that two agents have developed similar representations at some level. Furthermore, Pickering and Garrod (2004) suggest the same dynamics between the terms coordination and alignment as we do. First, the levels of coordination and alignment between two humans increase the more they interact. Second, coordination can be used as an indirect way of measuring alignment.

### 2.4 Formally defined dialogues among humans or computational agents

Several studies define goal-oriented human dialogues (Mann, 1988; Walton and Krabbe, 1995), or agent communication in OMAS (Hulstijn et al., 2005; Dignum et al., 2001; Atkinson et al., 2012; Prakken, 2009), usually using formal frameworks, describing allowed messages, information exchange, or action commitment.

#### 2.4.1 Descriptions of human dialogues

Consequently, different types of dialogue are described, using human conversations as examples. The authors of Mann (1988) suggest that all human interaction can be described with their proposed “dialogue games:” a task-oriented interaction between two people. Specifically, the dialogue games are (i) turn-based, (ii) consist of an Initiator, and a Respondee (iii) both of which aim to pursue some goal. These dialogue games are what we describe in a more fine-grained manner in our “Interaction Components” (I1–I6). The authors define goal as the wanted outcome of the interaction, in terms of a subset of states that each agent prefers to be. Therefore, their goal is best described by our I1. “Interaction Task.” Instead, what we define as P1. “Goal” aims to reflect the broader pursuit of establishing shared understanding, and not only to perform a particular task. Additionally, in Mann (1988), the humans seem to already have an established shared, task-oriented understanding, which allows them to successfully interact within dialogue games. Furthermore, our P1. “Goal” is described as something that is usually achieved over multiple interactions, where the I1. “Interaction Task,” is a guide or an indicator toward the goal achievement.

#### 2.4.2 Dialogue systems: formalizations of commitment-oriented agent interaction

Based on the studies of Walton and Krabbe (1995), researchers have also put forward the definition of “dialogue systems” to formalize computational agent interaction (Dignum et al., 2001; Atkinson et al., 2012). Specifically, in Dignum et al. (2001) formal dialogues are proposed that formally define what type of message can follow the last message. These dialogues allow the agents to exchange information, intentions, and commit to actions, enabling the controlling agent to form a team and devise cooperation plans. In Atkinson et al. (2012), the difference between deliberation and persuasion dialogues is put forward. In both cases, a group of agents need to collectively make a decision. In the former case, the agents need to communicate their own needs and world beliefs, forming a common set of requirements that need to be satisfied. In the latter case, the agents do the same, but without needing to agree to a common set of criteria. Instead, each of them communicates their own needs. The difference between these two types of dialogues is formally defined in Prolog rules, where different dialogue types have messages with different pre-conditions and post-conditions.

#### 2.4.3 Frameworks describing dialogues among computational agents

In Prakken (2009), another framework inspired by Walton and Krabbe (1995) is presented, that structures agent interaction and can calculate the outcomes of the dialogue at each step in terms of commitments. It also has constituents similar to our components. A link between components from our framework and the corresponding ones from Prakken (2009) is presented in Table 1. The “participants” are the initiator and respondee agents in our case. Its “topic language” and “communication language” allow the agents to understand each other's messages and, together with the “context” that consists of static and presupposed common knowledge, are part of our P4. “Prior Shared Understanding.” Similarly, the “outcome rules” that interpret the conclusion of a dialogue are also part of our P4. “Prior Shared Understanding.” The “effect rules” are updating a common set of commitments, based on the last message of the dialogue and the current commitments. “Effect rules” are similar to our A2. “Understanding Update” in terms of message outcome, the important difference is that the “effect rules” are the same for all agents, while the A2. “Understanding Updates” are personal for each agent, who decides privately what is the learning outcome of each interaction. Additionally, the learning outcomes can differ for the two interacting agents. The “dialogue purpose” depends on the type of dialogue. It can be conflict resolution regarding a specific proposition in case of persuasion, or resource allocation in case of negotiation. It is similar to our I1. “Interaction Task” and the same as our P1. “Goal” that describe the purpose of individual interactions, and what outcome they want to have over multiple interactions, respectively. The framework also has a “protocol” formally defining what messages can follow an ongoing dialogue. Our communication acts (I3–I4), i.e., Initiator's, Respondee's, and Concluding Act's, are an informal version of “protocol,” defining all possible acts, but not allowed act successions, nor define them formally.

**Table 1:** Some components of the framework we put forward, together with semantically corresponding components of earlier studies' frameworks (Hulstijn et al., 2005; Prakken, 2009).

**Our component**	**Description**	**Component in Hulstijn et al. (2005)**	**Component in Prakken (2009)**
P1. Goal	Intended Outcome?	-	Dialogue Purpose
P4. Prior Shared Understanding	What the agents already understand in the same way?	1. Initial context, 3. Combination rules	Topic Language, Communication Language, Context, Protocol, Outcome Rules
I2. Interaction scenario	Initial state of interaction	1. Initial Context	-
I3. Initiator's act, I4. Respondee's act, I5. Concluding act	Communication actions	2. Dialogue Acts	Protocol
**A2. Understanding Update**	Updates on internal representations	4. Update rules	Effect rules

Relations are not one-to-one. “A2. Understanding update,” is in bold denoting weaker connection, since our component refers to each agent performs independently, based on its own subjective interpretation of the interaction. Instead, “4. Update rules” and “Effect rules” describe commonly perceived and accepted outcomes across participants.

Similarly, the authors of Hulstijn et al. (2005) have applied the aforementioned abstract framework of Mann (1988) to formally describe interaction between artificial agents. In more detail, their framework aims to assist on verifying successful communication among agents. They suggest that most communication protocols can only perform verification on the form of the messages and not on their meaning. The authors aim to make the agent behavior more constrained following their suggested meaning-based coherence constraints. This way, the agents can ensure semantic verification while not having to follow a very strict communication protocol. In this study, the agent interaction is defined again as a dialogue game consisting of components. We will describe these components while showing their relation with our components. A summary of how the components relate is presented in Table 1. “Initial Context” defines the expectations and commitments of the agents regarding their dialogue, i.e., our P4. “Prior Shared Understanding” while also the initial state of the interaction, i.e., our I2. “Interaction Scenario.” “Dialogue acts” capture the agents' communicative action vocabulary, represented in our communication acts (I3-I5). “Combination Rules” represents the possible or mandatory actions for a given dialogue state that is reflected in our P4. “Prior Shared Understanding.” “Updated Rules” shows what commitments the agents make by performing a particular dialogue act. Instead, we propose A2. “Understanding Update,” which updates the current understanding of the agent regarding the other agents, the communication acts, and possibly the task, after an interaction cycle. Last but not least, “The End Contexts” signals the termination of a dialogue, similar to our I5. “Concluding Act.” Same as this study, our protocol does not come with strict definitions, but is aimed to be used as a resource while designing agent policies. The main difference with this study is that in Hulstijn et al. (2005), the agents have a static understanding of each other and their task, and only focus making public commitments and coordinating. In contrast, in our framework achieving the task or coordinating is not the end-goal, and the interaction is just a means to establish and verify shared understanding. The additional difference is that our framework takes into account that some agents might be humans.

#### 2.4.4 Differences of aforementioned dialogues and establishing shared understanding

In this section, we presented studies of formal representations of agent dialogues that enable them to successfully communicate in an OMAS setting, in order to exchange information, negotiate over action commitments, or resolve conflicts over propositions. In the aforementioned studies, the agents engage in formally defined, commonly interpreted and understood interactions. Thus, these approaches take a shared (task-oriented) understanding as a precondition for successful interaction. Instead, in line with Atencia and Schorlemmer (2012), we propose a framework that describes the inverse process: How two agents can establish semantic similarity, i.e., shared (task-oriented) understanding, using (successful) interaction as the guiding signal. In more detail, the framework describes agent interactions where semantics are not shared and presupposed, allowing room for ambiguity, misunderstanding, and personal interpretations that are not necessarily the same. We believe that it is necessary to focus on such scenarios when addressing hybrid populations, as humans should not be expected to apply or interpret formal semantics to engage in such dialogues as computational agents do. Our framework aims to acknowledge that each agent (human or artificial) has private interpretations that cannot always be accessed, interpreted, or verified. Subsequently, it describes a process through which such agents can learn to understand each other.

### 2.5 Establishing shared query understanding in an open multi-agent system

Our study is in line with earlier work presented in Kondylidis et al. (2023), where the agent interaction is defined as follows: A Teacher agent needs the Student agent to perform an action. To explain that action, the Teacher provides examples (I3. “Initiator's Act”). The Student agent performs actions to the environment (I4. “Respondee's Act”), with observable outcomes (I3. “Concluding Act”), that allow the Teacher to estimate how well its examples were understood (A3. “Understanding Evaluation”). These interaction-outcome cycles allow the Student to incrementally understand (A2. “Understanding Update”) what the Teacher wants them to do. The study presents three restrictions that need to be taken into account for establishing shared task-oriented understanding in a hybrid OMAS. We further analyze these restrictions and say which ones are needed for all OMAS cases to ensure the establishment of shared understanding and which are only necessary for the special hybrid OMAS case. The ones that are needed for all OMAS cases are pointed out in the definition of our framework, and specifically on the components that are related. One of them is that there must be a Prior Shared Understanding in terms of agent expectations, commitments, or some provided interaction protocol, that the agents can use to further extend their shared understanding through interaction. The other one is that Task Performance must reflect levels of shared understanding if external validations of agents' (shared) understanding is not possible, due to lack of accessibility or tools to interpret. The restrictions that are only required for the hybrid OMAS special case are presented in Section 3.4, which are the following. Restriction 1: the communication needs to be physical and concise in terms of volume or comprehension demands. Restriction 2: the task performance must reflect levels of shared understanding or goal achievement, since the internal representations of a human are not accessible or interpretable. Restriction 3: the evaluation of a proposed method must include its efficiency (i) in terms of duration, since human participants are expected to be available for a limited time, while preferably also (ii) including estimates of cognitive effort needed from the side of the human participant.

## 3 The shared (task-oriented) understanding establishment framework

In this section, we provide a framework that describes how agents can establish shared (task-oriented) understanding. To assist us with our term definition, we first present an example where a travel agent communicates with a client that aims to find a preferred trip destination. Then, we put forward our definitions of task-oriented understanding and shared (task-oriented) understanding. We finally describe the process through which two agents can establish and evaluate shared (task-oriented) understanding in the form of a framework.

### 3.1 An example of a travel consultant establishing shared (task-oriented) understanding with a client

A person wants to organize their next holidays, but is very busy to do their own research. They decide to use a travel agency service that charges per minute of use. The former will be referred to as the **Client**, while the latter as the **Consultant**, that can be a person or a chatbot. Both should be perceived as interacting agents that aim to establish shared (task-oriented) understanding, i.e., understand each other well enough to find a suitable vacation plan for the Client. The client wants the trip to conform to some criteria, that they vaguely define. For example, they are looking for a vacation plan that is “*low cost*,” “*comfortable to get there*,” and “*close to a hospital*” due to a recent health implication. The Consultant has access to a collection of holiday plans, but has to disambiguate the Client's criteria and for example define “*low cost*” in terms of a specific price range, or correctly interpret what the Client perceives as a “*comfortable way to travel*.” The two agents interact over natural language in order to explain to each other the criteria or the possible options, so that a suitable vacation plan can be selected. Their interactions are criteria-focused. Specifically, the Client provides a description for each criterion, i.e., “I do not like flying” to the Consultant, who in turn tries to propose a holiday plan that complies with this criterion. Then, the Client replies by expressing if this criterion is satisfied in the proposed holiday plan. If it does not, the client provides another explanation of its criterion, i.e., “I also do not like taking the bus.” In case the criterion is satisfied, they move to the next criterion description. The Client is charged per minute of interaction, aiming to find a good vacation plan fast.

### 3.2 Defining individual and shared (task-oriented) understanding

In this work, we focus on specific cases of agent understanding that can be observed or measured, even in an indirect manner. We propose the following three terms:

- Individual task-oriented understanding: the understanding of a single agent required to perform a particular task.
- Shared task-oriented understanding: the common understanding of a group of agents required to perform a task that demands them to cooperate.
- Shared understanding: a group of agents that can refer to each other's similar internal representations.

Here, we will describe these terms in detail and ways to achieve them. The term of shared understanding, as a similar enough interpretation of communicated symbols by a group of agents, has also been mentioned by Atencia and Schorlemmer (2012), while in Pickering and Garrod (2004) it is mentioned as “alignment” referring to human agents. In the following definitions we aim to separate it from shared task-oriented understanding, which only considers similar enough term interpretation between agents, conditioned to performing a particular task, referred to as “coordination” in Pickering and Garrod (2004).

#### 3.2.1 Individual task-oriented understanding

We define individual task-oriented understanding as the ability of an agent to choose its actions in order to successfully perform a particular task in an environment. Directly evaluating the task-oriented understanding of an agent is challenging, as there can be several ways to perform a task, making it hard to evaluate them or even to calculate all of them. Additionally, we might not have access to the internal representations of an agent or might not be able to interpret them. This is the case for natural agents, i.e., humans, and for artificial agents that use black-box methods, like deep neural networks. For this reason, we measure task-oriented understanding indirectly through the task evaluation metrics. In our example, both the Client and the Consultant already have individual task-oriented understanding, as either of them could select the best vacation plan, had they both access to the criteria and the possible options.

#### 3.2.2 Establishing task-oriented understanding

Learning is an incremental process in which an agent can extend or develop its task-oriented understanding when provided with (new) interaction episodes. Throughout this process, the agent develops an understanding of its actions and the environment, as well as how they affect each other with respect to achieving the task. An example of methods that allow such development of task-oriented understanding are Reinforcement Learning (RL) methods. Since learning is an incremental process, the evaluation of such methods is usually coupled with efficiency evaluation metrics, e.g., number of interactions, and is presented in the form of a trade-off between task performance and the costs needed to achieve it.

#### 3.2.3 Shared task-oriented understanding

Some tasks require the agents to exchange information in order to perform the task, like in our example. We define that two agents have established shared task-oriented understanding when they can perform a task that requires their cooperation. Again, internal agent representations might not be accessible, interpretable, or might be defined in different semantic spaces. This restricts us from directly evaluating how well the agents understand each other, and leads us to once again indirectly measure shared task-oriented understanding based on task performance. An example of establishing shared-task oriented understanding is language games (Steels and Loetzsch, 2012). It is important to make sure that the task requires agent cooperation, as pointed out in Kondylidis et al. (2023). Differently, task performance could reflect levels of task-oriented understanding for each individual agent, and not shared. Task cooperation is required in our example, as neither agent can alone select the best vacation plan for the Client. Additionally, it should be noted that established shared task-oriented understanding does not imply the same task-oriented understanding across the individual agents. In our example, the Consultant might have different criteria for a good vacation plan than the Client. Instead, shared task-oriented understanding suggests that the agents interpret the communication signals in a similar enough manner, that allows them to perform the downstream task, as pointed out by Steels and Loetzsch (2012); Laera et al. (2007). In our presented case, the agents do not need to agree on what “low cost” means down to the cent, but only have to place them in roughly the same range. This is the reason why even heterogeneous agents with different task-oriented understanding can still communicate successfully to perform some task. In our example, a satisfied Client is evidence of shared task-oriented understanding between the Client and the Consultant.

#### 3.2.4 Shared understanding

The agents can use their interaction episodes to establish a shared understanding that goes beyond performing the task. Our definition is in line with Atencia and Schorlemmer (2012), where the ultimate goal of the interaction is not necessarily limited to successfully perform the task. Instead, a number of interaction episodes are used so that the agents can eventually establish similar enough semantic interpretations or representations, i.e., a shared understanding. The goal, that requires shared understanding, can be the same as the task, as is the case in Kondylidis et al. (2023). In other cases, the interactions do not necessarily have a particular task, or at least that task is not the main focus, but aim to establish a shared understanding that transcends single interactions. This is the case for Laera et al. (2007), where the agents interact following a provided formal negotiation framework, that allows them to align ontology terms, i.e. establish shared understanding, after a number of interactions. If the goal is not in line with the task, the goal of achieving shared understanding cannot be indirectly measured using task performance as a signal. In that case, the agents' shared understanding should either be evaluated by asking them to perform some other task, or can be compared to some ground truth shared understanding, i.e., known ontology alignments. The latter is not always possible, as it would require the agents' internal representations to be both accessible and interpretable. In our example, where the goal is to find a good vacation plan for the Client, the task for each interaction within their communication is to satisfy a specific criterion. Toward this goal, the agents also establish shared understanding regarding the Client's ambiguous criteria, i.e., “*low cost*.” As put forward by Rovatsos et al. (2003), we also support the idea that the meaning of an utterance cannot be disentangled by the context in which it is used. Notice that the agents in our example have established shared understanding of “*low cost*” regarding vacation plans, and may still have a different opinion of what “*low cost*” is for a different task, i.e., purchasing a car.

#### 3.2.5 Establishing shared (task-oriented) understanding

Shared task-oriented understanding can also be extended or developed incrementally, provided some task-oriented agent interaction episodes. The agents must have some prior shared-understanding, i.e., common vocabulary, similar environment perception etc., that is then further aligned or extended either to directly establish shared understanding or indirectly do so by establishing shared task-oriented understanding. In our example, the interacting agents speak the same language and further interact to find a good vacation plan for the Client, i.e., establish shared task-oriented understanding, which requires them to first align their perception on acceptable criteria values, i.e., establish shared understanding.

### 3.3 Proposed framework

**Figure 1:**
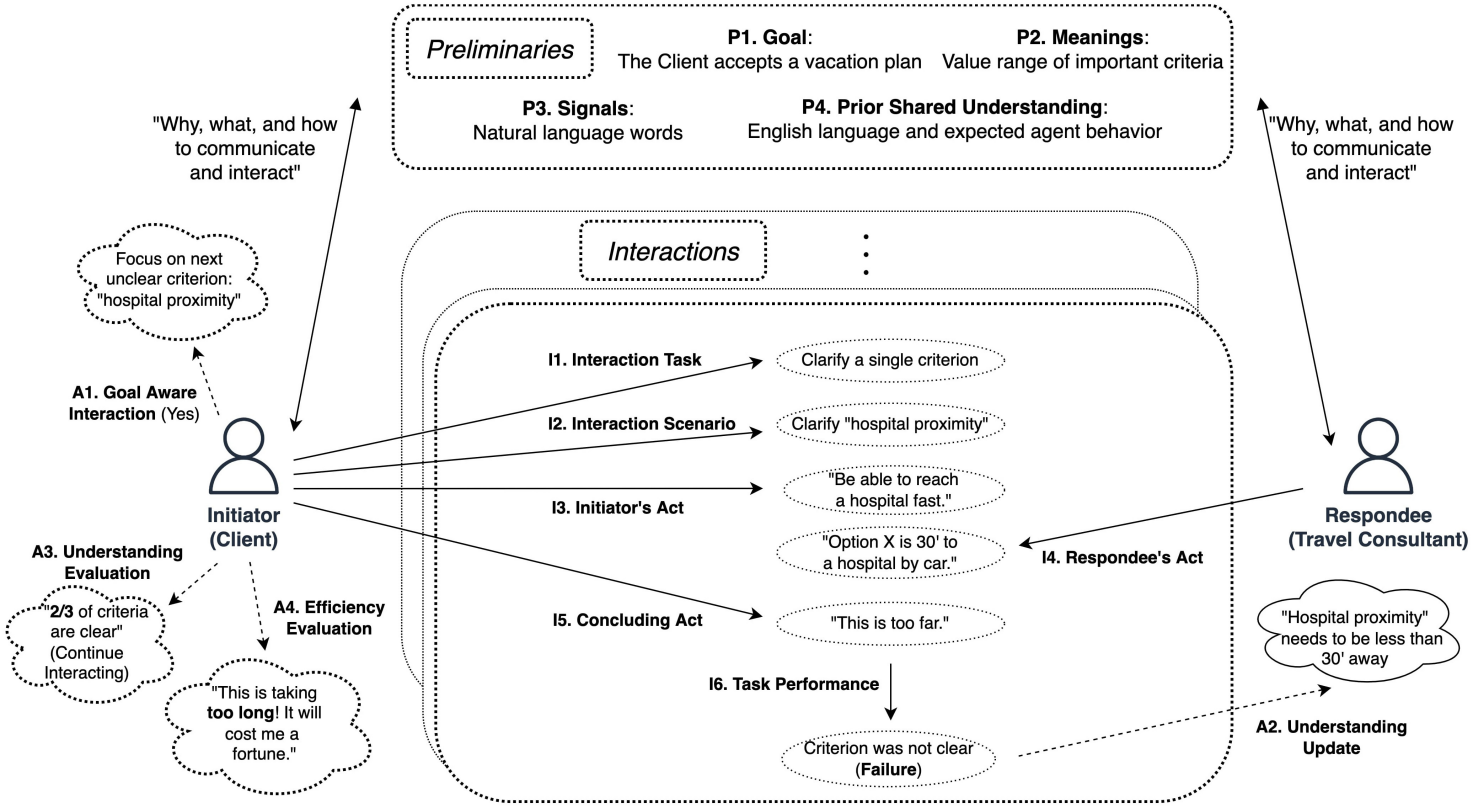
A depiction of how our framework captures the interaction between the Client and the Consultant from our example, while being on the process of understanding each other well enough and help the Client choose a vacation plan. At the **top**, we see the Preliminary components (P1–P4). In the **middle**, we see the Interaction components (I1–I6) describing one of their interactions, and on the **sides** we see the Agent components (A1–A4). The component definitions are in Section 3.3.

Our framework aims to structure the process of designing agents that establish shared (task-oriented) understanding in a hybrid OMAS. The framework is defined in terms of components, or questions that one needs to answer when designing an agent for such a system. To apply the framework for a specific use-case, the agent designer needs to answer the questions of each component. As a demonstration, we answer the question of each component for the example presented in Section 3.1, at the end of each component's definition. There are three component categories. The *Preliminary components* (P1–P4) describe what is the shared understanding goal, what and how the agents can communicate, and what they already understand in common. The *Interaction components* (I1–I6) define the task, the possible actions and the outcomes of a single interaction. The *Agent policy* components (A1–A4) define how the agent's behavior is affecting the interaction, and how the agent's understanding is updated and evaluated after each interaction toward the goal of shared understanding. In this study, we focus on the abstract case of interactions between pairs of agents, to allow generalizability of our framework to larger populations and more complex interactions. Figure 1 illustrates how our framework structures the process of establishing shared understanding for the case of our Client-Consultant example.

#### 3.3.1 The preliminary components (P1–P4)

We will first revisit the description of the shared understanding goal, as stated in Section 3.2.4, now as a framework component (P1). Then, define what concepts are involved in the interaction (P2), how the interaction messages look like (P3), and what can be used as common communication ground (P4) (Keysar et al., 1998), from the interactions to be meaningful.

##### 3.3.1.1 P1. Goal

“*What is the Goal that requires the agents to communicate?*” The agents interact in order to achieve some shared understanding goal. This means that they want to be able to develop or align some meaning representations. The representations do not need to be identical, but only similar enough. The goal can be the same as the interaction task, but it can also differ. In our example, the goal is for the Client to select the preferred holiday plan.

##### 3.3.1.2 P2. Meanings

“*Which concepts, i.e., internal representations, are related to the goal, that the agents should be able to communicate successfully?*” Depending on what the agents want to achieve together, different meanings or concepts can be involved. It is important to clearly define which concepts the agent will want to be able to communicate. In our example, these concepts are “*low cost*,” “*comfortable to get there*,” and “*close to a hospital*.”

##### 3.3.1.3 P3. Signals

“*What signals can the agent create?*” Agents cannot directly communicate concepts, but need to implicitly do so by relating them with signals, the same way a word has a meaning and a form. Defining the signal space depends on the physiology of the agents, as well as on the environment. Does the agent have a physical body and cameras? Is the environment dark or noisy? The agents need to know which set of signals are perceivable by both participants, since being able to communicate requires a successful mapping between meanings and signals. In our example, the agents use words for signals, allowing them to put together messages that consist of sentences.

##### 3.3.1.4 P4. Prior shared understanding

“*What do the agents already understand in the same way?*” Establishing shared understanding is an incremental process and requires some common understanding to already exist and act as a common ground (Keysar et al., 1998). This can be for example in the form of common representations, similar experiences, common hypotheses about the communication acts, an elementary communication or negotiation protocol, and so forth. This is defined as the “Grounded Communication” restriction in Kondylidis et al. (2023), in the sense that there exists a set of symbols or signals that both agents identify in the same or similar way, prior to interacting, e.g., URIs representing identities of people or other entities in Kondylidis et al. (2023). In our example, we assume that the agents speak the same language. Although natural language might include ambiguities, like the “low cost holiday plan,” we assume most words to be interpreted in the same way. The agents also share an understanding of what the goal is and what are the agents roles and expectations within an interaction.

#### 3.3.2 The interaction components (I1–I6)

Once the preliminaries are clear, the interaction ground rules and expectations must be set. We focus on directed interactions between two agents, i.e., the Initiator and the Respondee, aiming to design an abstract interaction schema that can then be extended to include more agents and different types of communication such as broadcasting. The agents interact around performing a specific task before the end of the interaction. Their performance on the task should act as an indicator on how well the agents understood each other to the extent that the task required, while also what to learn from this interaction. In our example, the Initiator is the Client and the Respondee is the Consultant. Each interaction is centered around a single criterion. It begins with the Client uttering a description of the criterion, the Consultant proposes a holiday plan accordingly and concludes with the Client expressing its satisfaction or rejection. In case of the latter, the Client provides another description of its criterion.

##### 3.3.2.1 I1. The interaction task

“*What do the agents try to do within each individual interaction?*” Agent interaction is structured around a task that can be performed and potentially evaluated within individual interactions. In case we want to use the Interaction Task to indirectly measure the levels of shared (task-oriented) understanding of the interacting agents, it is imperative that the task requires agent cooperation in order to be performed; the “Cooperation” restriction defined in Kondylidis et al. (2023). Additionally, the interaction task can be the same or in line with “Goal” defined in Section 3.3.1, in the sense that enough task interactions should allow the agents to achieve their shared understanding goal. In our example, the task is to agree on the interpretation of the Client's search criteria, regarding a single criterion.

##### 3.3.2.2 I2. Interaction scenario

“*Does something make this particular interaction different from others, apart from agent behavior?*” Within an interaction and before the agents begin to perform communication acts, there might be some initial information already provided to (some of) them. Put differently, the interaction scenario is not the same across all interactions, and affects the agents' decisions when selecting their communication acts. This can sometimes be empty or open, allowing the initiator to shape the direction of the interaction. Differently, it can be (randomly) provided by the environment. In our example, the interaction scenario is open, allowing the Client to decide which criterion to further clarify during each interaction, e.g., what “*close to a hospital*” means to them.

##### 3.3.2.3 I3. Initiator's acts

“*What kind of messages does the initiator of the interaction send?*” In our example, the Client, acting as initiator, utters a criterion that is important to them, and attempts to explain it using messages in natural language, e.g. “I want to be able to reach a hospital fast.”

##### 3.3.2.4 I4. Respondee's acts

“*How does the Respondee reply?*” An example response message from the Consultant could be: “The holiday destination X, is only 40 km away from a hospital and you can drive there within 30 min.”

##### 3.3.2.5 I5. Concluding act

“*What event or communication act indicates the termination of the interaction?*” Is this signal coming from the Initiator, the Respondee, both, or the environment? Usually, this signal contains or allows the calculation of the agents' performance on the task. In our example, the Initiator would conclude the interaction, either by saying his criterion is satisfied and the hospital can be reached fast enough, or say that this is not close enough.

##### 3.3.2.6 I6. Task performance

“*How is the task performance measured and in what evaluation metrics is it defined in?*” It can also be the case that the interaction does not come with a measurable task performance. For example, a meaning negotiation interaction might not provide a new agreed alignment, but this does not necessarily imply bad communication nor is a case of a backward step toward achieving shared understanding. It should be reminded that, if we want task performance to reflect levels of shared (task-oriented) understanding, then high performance should only be possible if the agents exhibit cooperation, as the “Cooperation” restriction in Kondylidis et al. (2023) suggests. In our example, the task performance can be represented in a boolean manner reflecting the Client's satisfaction. The task performance is in line with the goal and reflects goal performance, since disambiguating a single criterion helps toward selecting a good vacation plan. At the same time, agent cooperation is required to perform the task since the Client understands the criteria while the Consultant can provide matching holiday plans, but neither of them can perform the task alone. Therefore, task performance reflects levels of shared understanding in our example.

#### 3.3.3 Agent policy components (A1–A4)

After structuring the interaction between the agents, we can now see where the agent policies, i.e., the agent decisions, come into effect.

##### 3.3.3.1 A1. Goal-aware interactions

“*Is the agent aware that the current interaction aims to help them achieve some broader shared understanding Goal? If so, is that affecting its behavior within individual interactions?”* For example, a more goal-aware communication policy may interleave between exploring signals with uncertain meaning and sacrificing task performance in a particular interaction, or to exploit signals with known meaning as to achieve higher task performance. In our example, the Client decides which criterion to describe in each interaction, making it an example of goal-aware agent policy.

##### 3.3.3.2 A2. Understanding Update

“*How should the agent use the interaction outcome to learn?*” The interaction components form an interaction episode, from which at least one agent should be able to increase its understanding. The Understanding Update policy allows the agent to figure out what to learn, i.e., which interpretation assumptions to reject or reinforce. In our example, the Consultant updates its understanding of the Client's criteria by further narrowing down the range of their acceptable values after each interaction.

##### 3.3.3.3 A3. Understanding evaluation

“*How can we measure the levels of agents' shared understanding?*” The understanding evaluation policy allows the agent to estimate how well it understands or can be understood by other agents, with respect to the shared understanding Goal (P1). It can be the same as the interaction task performance, or its aggregation over multiple interactions, or even require external validation. In case of the former two cases, high task performance should only be possible if the task requires cooperation, in line with the “Cooperation restriction” in Kondylidis et al. (2023). An intelligent agent policy, that is goal-aware and has access to this evaluation, can use it in order to decide how to behave in the next interaction. As the main way to measure the levels of desired shared understanding, it can also be used to decide when to terminate the agents' interactions. In our example, the shared understanding is reflected by the task performance, in terms of numbers of satisfied criteria. When all criteria are satisfied, the Client stops interacting with the Consultant. Differently, it can decide which criterion to further attempt to disambiguate in the next interaction.

##### 3.3.3.4 A4. Efficiency evaluation

“*At which cost have the agents reached the current levels of shared understanding?*” Efficiency can be measured in terms of number of interactions (common cost across agents), but also for example by estimating the effort or energy needed by each participating agent separately, e.g., depending on their physiology. In our example, the cost of the interaction depends on its time duration.

### 3.4 Establishing shared (task-oriented) understanding in hybrid populations

So far, we have described the process of establishing shared (task-oriented) understanding in OMAS, and mentioned any required restrictions according to Kondylidis et al. (2023) for that setting. Here, we follow the assumption that hybrid populations can be studied as a special case of OMAS. Accordingly, we point out the restrictions put forward by Kondylidis et al. (2023) that affect the hybrid OMAS special case, and see which components of our framework are affected by each of them. This aims to be used as a tool to assess the compatibility of an OMAS study to hybrid populations, and to help identify what adaptations might be necessary for such an application.

#### 3.4.1 Restriction 1: physical and concise communication

For the case of human participants, P3. Signals can be affected, since a human participant can confine the types of interpretable signals to ones that have physical form or affect, i.e., sound, unlike an unrendered digital message. Most importantly, in a hybrid population, the agent's messages must be concise enough to be understood or generated by a human without requiring too much effort. This restriction affects the Communication Acts (I3–I5), and mostly restricting them in terms of volume, i.e., comprehension effort, or compactness, i.e., interpretation effort. In other words, the messages, i.e., the Communication Acts (I3–I5), should be short and self-explanatory.

#### 3.4.2 Restriction 2: task performance reflects shared understanding

For the special case of hybrid populations, it is impossible to directly evaluate the agents' shared understanding, since the internal representations are not accessible or interpretable, since they are brain activations. Therefore, it is necessary that the P1. Goal is in line with the I1. Interaction Task, so that I6. Task performance can allow us to indirectly evaluate levels of shared understanding, i.e., P3. Understanding evaluation.

#### 3.4.3 Restriction 3: efficiency is evaluated

Given enough interactions, two agents can eventually even understand perfectly each other, but when a human is in the loop, we must be cautious with their time. Subsequently, it's not only a matter of achieving high levels of shared understanding or high task performance, but doing so as far as it is useful for the task. Therefore, when evaluating a method, the A3. Understanding Evaluation must be reported with respect to the P4. Efficiency Evaluation, that has to be somehow measured, estimated, or even reported by the human participant.

## 4 Applying our framework on existing studies

In this section, we aim to answer our three research questions, using our framework as a tool to analyze 8 relevant studies. We first provide an overview of the studies that will be analyzed, and we explain their selection. Then, we analyze these studies in terms of our framework, in order to answer our first research question: “Can we represent the process of establishing shared understanding in a framework?” As a next step, we use our framework to determine what types of understanding are provided or established in each study, answering our second research question: “Can this framework help us identify whether two agents indeed establish shared understanding.” Finally, we evaluate the applicability of these studies to hybrid populations, following the definitions and restrictions of our framework, as to answer our third research question: “Can this framework allow us to foresee limitations of existing studies if they were to be applied in hybrid populations?”

### 4.1 Overview of the analyzed studies

The analyzed studies come from the domains of query answering (Kondylidis et al., 2023), language games (Steels and Loetzsch, 2012), human-agent task tutoring (Mohan and Laird, 2014), agent-based ontology alignment (Euzenat, 2014; Laera et al., 2007; Atencia and Schorlemmer, 2012; Anslow and Rovatsos, 2015), and reward driven OMAS (Anslow and Rovatsos, 2015).

In Kondylidis et al. (2023), the authors describe how a human user can query a database without having to first familiarize with its schema. The user uses items of the database that they are familiar with, to give examples of what the query describes and what it does not. This allows the agent that represents the database to incrementally approximate the query using its schema. Because the user does not know the answers to their query, they cannot evaluate them themselves. The answers are provided to an expert that can evaluate them and inform the user to what extent their query was correctly interpreted by the database agent.

Language games (Steels and Loetzsch, 2012) experimentally prove how a population of agents can come up with a communication language, based on task-oriented pairwise agent interactions. The agents need to develop a new vocabulary of concepts and align its interpretation to be able to refer to objects in their environment. In the Non-Grounded version, the agents can already identify the objects and need to figure out how to use a common vocabulary to refer to them: they focus on establishing shared task-oriented understanding. In the Grounded version, the agents need to also come up with object descriptions at the same time, being able to distinguish each object from a group of objects: they also develop individual task-oriented understanding at the same time. Words are interpreted in the space of object characteristics. Agents interact in pairs and specifically in a “speaker-listener” setting, where the former acts as tutor and indirectly defines the meaning of the used word, allowing both of them to update the word interpretation based on similar observations and toward a common convergence.

In Mohan and Laird (2014), the agent is learning from a human tutor how to decompose complex tasks and perform them in the environment using a robotic arm. The tasks require the manipulation of small foam blocks on a table-top workspace that simulates a kitchen. The human tutor asks the agent to perform a task and the latter either performs it or asks for instructions, i.e., its decomposition to known tasks. Through this process, the agent learns to (i) decompose tasks to subtasks that it already knows and (ii) what parameters are implied for specific tasks (the “store” action always has “pantry” as a location).

We will also apply our framework on 4 interaction-based ontology alignment studies (Euzenat, 2014; Laera et al., 2007; Atencia and Schorlemmer, 2012; Anslow and Rovatsos, 2015), since these also study how two agents that represent knowledge or information differently can still understand each other. In Euzenat (2014), the authors apply an adapted version of language games. Specifically, the agents describe common instances to each other, using properties from their own schema. Eventually, the agents create or refine schema alignments. In Laera et al. (2007), agents have access to a publicly accessible set of symbol alignments. They can follow a formal argumentation framework to suggest or attack symbol alignments based on facts or preference scores, that each agent calculates based on their ontology or on heuristic values produced by comparing parts of their ontologies. In Atencia and Schorlemmer (2012), a pair of agents engage in a conversation that represents the formation and the answer of a query from each party. The agents are provided a shared language, and each of them has its own internal automaton representing the discourse model of the conversation. Through interaction, they figure out further symbol alignments across their personal ontologies. The interaction success is assumed if both agents reach a terminal state in their automata on the same step of the conversation, as a simplified version of actually evaluating the query answer. In Anslow and Rovatsos (2015), a group of agents that have a common schema want to exchange environmental observations. The observations are from sensors that are placed in static locations. The agents move around the environment and broadcast measurements of these sensors when they are in their proximity. Each agent gives a different name to each sensor, that they try to align over time, essentially solving an instance matching problem while aiming to minimize both the time needed as well as the amount of broadcasted information.

Finally, the authors of Rovatsos et al. (2003) describe a method where agents aim to maximize their personal reward, which is affected by the behavior of other agents. The agents can communicate symbols that represent action commitments and interpret them based on the agent behavior that follows. The agent policy aims to both maximize returned reward and minimize symbol interpretation uncertainty, applying an exploration-exploitation method to do so.

### 4.2 Purpose of the selected analyzed studies

These 8 studies are selected to provide diverse and computationally defined examples of the shared (task-oriented) understanding terms and the components of the framework. Additionally, the examples are intended to stress how iterative task-oriented interactions can overall build up toward a goal which may be the same or in some cases transcend the interaction task. In Kondylidis et al. (2023); Anslow and Rovatsos (2015), the interaction task and the goal are aligned, making it simple cases where the agents iteratively interact over the same task until they reach high enough task performance. In both cases, the agents do not interact in order to produce semantic alignments, and this happens as a side effect. Furthermore, in Steels and Loetzsch (2012); Mohan and Laird (2014); Euzenat (2014); Laera et al. (2007); Atencia and Schorlemmer (2012), the agents interact for some task that is different from their goal, i.e., to establish semantic alignments. Specifically, Steels and Loetzsch (2012) is a good example of a case where the I1 Interaction Task, i.e., reference game, is not directly linked to the P1. Goal. Simply put, it is not really about selecting the right objects, but the shared language that emerges over time as a side effect of this process. Additionally, Steels and Loetzsch (2012) experimentally demonstrates that agent interpretations only need to be similar enough to allow the agents to communicate successfully and perform the task. This serves as evidence that shared task-oriented understanding can and should only be measured indirectly using task performance, and not by comparing internal representations of agents directly. In Mohan and Laird (2014), the human wants to teach the agent how to perform a range of tasks, while also what should be assumed for each task. This study was selected as an example of establishing shared (task-oriented) understanding between a human and a robotic agent. Additionally, after enough examples, the agent can infer common assumptions about specific tasks, while those are not mentioned by the human. In Euzenat (2014); Atencia and Schorlemmer (2012) the ultimate goal is to establish OA, although the agents perform this by engaging in different I1. Interaction Tasks. In Euzenat (2014), the agents guess how the other agent would describe an object (common instance), while in Atencia and Schorlemmer (2012), the agents iteratively run queries through each other. In both cases, the agents do not directly align symbols nor meanings, but this is an indirect effect of their interaction. On the contrary, in Laera et al. (2007), the agents explicitly engage in formal negotiations of symbol alignments as an I1. Interaction Task, although their P1. Goal is to be able to perform queries together (service requests). Finally, Rovatsos et al. (2003) is a counter example of establishing shared understanding, or at least the ability to prove this according to our definitions. This is because the agents engage in games on non-pure common interest, and therefore while their task performance is increasing over time, we cannot argue that their symbol interpretations are converging to similar values.

### 4.3 Analysis of existing studies

Here, we evaluate the generalisability of our framework, answering our first research question: “Can we represent the process of establishing shared understanding in a framework?” Table 2 summarizes the application of the aforementioned studies according to the components of our framework. The analysis is ordered and categorized according to the framework's components and our research questions.

**Table 2:** Study analysis according to our shared task-oriented understanding establishment framework.

**Framework analysis**	**Kondylidis et al. (2023)**	**Steels and Loetzsch (2012)**	**Mohan and Laird (2014)**	**Euzenat (2014)**	**Laera et al. (2007)**	**Atencia and Schorlemmer (2012)**	**Anslow and Rovatsos (2015)**	**Rovatsos et al. (2003)**
**Preliminary components**								
P1. Goal	Querying	Language	Task	Alignment	Service	Ontology	Information	{No Shared
		Emergence	Tutoring	Refinement	Request	Alignment	Update	Goal}
P2. Meanings	Ontology	Visual	Perception	Ontology	Ontology	Ontology	Ontology	Future
	Concepts	Features	& Predicates	Concepts	Concepts	Concepts	Concepts	Actions
P3. Signals	Symbols	Sounds &	Natural	Symbols	Symbols	Symbols	Symbols	Symbols
		Postures	Language					
P4. Prior Shared	Common	Common	Language &	Common	Language &	Overlapping	Ontology &	Negotiation
Understanding	Instances	Perception	Perception	Instances	Alignments	Vocabulary	Perception	Language
**Interaction components**								
I1. Interaction Task	Querying	Object	Command	Description	Alignment	Querying	Information	Negotiating
		Referral	Execution	Prediction	Negotiation		Update	Behaviors
I2. Interaction	{Open}	Candidate	{Open}	Presented	{Open}	{Open}	Sensor	{Open}
Scenario		Objects		Object			Proximity	
I3. Initiator's Acts	Contrastive	Spoken	Command in	Request to	Alignment	Query	Sensor	Action
	Examples	Word	English	Describe	Argument	Request	Value	Request
I4. Respondee's Acts	Query	Object	Acts or Asks	Object	Alignment	Query	(Explication	Negotiating
	Results	Selection		Description	Argument	Results	Request)	& Acting
I5. Concluding Act	Environment	Initiator	Deduction or	{None}	Either Agent	Either Agent	(Initiator	Set
	Evaluates	Supervises	Indication		Withdraws	Terminates	Explains)	Duration
I6. Task Performance	External Query	Success	Always	Success	{Not	Success	{Not	Individual
	Performance	(Boolean)	Success	(Boolean)	Evaluated}	(Boolean)	Evaluated}	Rewards
**Agent policy components**								
A1. Goal-Aware	Yes	No	No	No	Yes	No	No	Yes
A2. Understanding	Private Query	Interpretation	Interpretation	Alignment	Update	Alignment	Instance	Expectation
Update	Approximation	Updates	Updates	Refinements	Alignments	Refinements	Alignments	Updates
A3. Understanding	Query	Smoothed	Autonomous	Combination	Alignment	Smoothed	Alignment	{Not Shared
Evaluation	Performance	Accuracy	Execution	of Metrics	Evaluation	Accuracy	Evaluation	Understanding}
A4. Efficiency	# of Examples	{Not	Number of	{Not	{Not	Number of	Communicated	{Not
Evaluation	& Memory Size	Evaluated}	Interactions	Evaluated}	Evaluated}	Interactions	Volume	Evaluated}
**Understanding establishment**								
Ind. Task-oriented	Provided	Established	Established	Provided	Provided	Provided	Provided	Established
Shared Task-oriented Und.	Established	Established	Established	Established	Provided	Established	Established	Not Est.
Shared Understanding	Established	Established	Established	Established	Established	Established	Established	Undetermined
**Hybrid compatible:**	YES	NO	YES	NO	NO	YES	NO	NO

The values of the components per study are analyzed in Section 4.3. The values of the lower rows regarding the types of understanding being Established or Provided are described in Section 4.4, and the Hybrid compatibility of the studies is commented in Section 4.5.

#### 4.3.1 Preliminary components

##### 4.3.1.1 P1. Goal

The agents iteratively interact with each other, in order to achieve a shared, task-oriented understanding. Toward what goal? In Kondylidis et al. (2023), one agent is trying to explain one particular query to the other agent. In Steels and Loetzsch (2012), a Language Games study, the agent interaction allows the emergence of a new language shared by all agents. This language allows them to be able to refer to world objects to each other. In Mohan and Laird (2014), the human tutor wants to teach some high level commands to a robot. In Euzenat (2014); Laera et al. (2007); Atencia and Schorlemmer (2012); Anslow and Rovatsos (2015) the goal is to perform ontology alignment. Specifically, in Euzenat (2014), the agents refine their existing concept alignments over time, by describing common instances to each other as a variation of Language Games methodology. In Laera et al. (2007), the agents want to be able to request services to each other, parts of which are defined in their respective ontologies. In Atencia and Schorlemmer (2012), the agents use their ontologies to perform queries to each other and over time they align concepts based on the success of their interaction. In Anslow and Rovatsos (2015), a population of agents aim to stay updated regarding the output of a set of sensors that are scattered around the environment. Finally, in Rovatsos et al. (2003), the agents do not have a shared understanding goal, but only aim to maximize their individual reward.

##### 4.3.1.2 P2. Meanings

Communication takes place using signals that each agent interprets by relating them with meanings. We will now see what are the meanings in each of the analyzed studies. In Kondylidis et al. (2023); Euzenat (2014); Laera et al. (2007); Atencia and Schorlemmer (2012); Anslow and Rovatsos (2015), the communicated signals are ontology concepts. These can either be part of A-box (facts) or T-box (terminology) (Baader et al., 2003). In Steels and Loetzsch (2012), the communicated signals are interpreted in terms of visual characteristics of world objects. In Mohan and Laird (2014), the human has a set of tasks in mind and how the environment should look like after their execution. The robot is provided with tools to perceive the environment visually and spatially, while also some predicates representing known low-level tasks, and models to estimate their affect on the environment. In Rovatsos et al. (2003), the words are interpreted as commitments over future actions.

##### 4.3.1.3 P3. Signals

In most of the presented studies, the agent communication is happening over digital media (Kondylidis et al., 2023; Euzenat, 2014; Laera et al., 2007; Atencia and Schorlemmer, 2012; Rovatsos et al., 2003; Anslow and Rovatsos, 2015). Therefore, the signals have the form of discrete symbols, which can be used to compose more complex messages. This is different for Steels and Loetzsch (2012), where the agents communicate via the physical environment, using either sounds to pronounce made up words, or body postures to point to physical objects around them. In Mohan and Laird (2014), the agents communicate over natural language using a chat interface. Therefore, the signals are English words.

##### 4.3.1.4 P4. Prior shared understanding

The agents need to have some existing prior shared understanding that can act as common ground (Keysar et al., 1998), allowing them to further understand each other based on interaction experiences. In Kondylidis et al. (2023); Euzenat (2014), agents are provided with the ability to refer to a set of common instances that they are both aware of, although their knowledge about these instances can differ. In Steels and Loetzsch (2012), the agents are provided with a similar enough sensory perception, that allows them to relate words with similar enough visual characteristics. In Mohan and Laird (2014), the agents already understand a large enough part of the English language, allowing them to further align more words that refer to tasks. Additionally, they perceive the environment in a similar enough way, allowing them both to relate tasks and actions with world states. In Laera et al. (2007), the agents are provided a common negotiation framework, i.e., a common language to communicate, as well as a common public repository of concept alignments that allow them to negotiate about new or existing concept alignments. In Atencia and Schorlemmer (2012), the agents are sharing parts of their ontology and aim to align the remaining parts after enough interactions. In Anslow and Rovatsos (2015), the agents are observing the sensors of the environment in the same way, but they have to be close enough to them. Their knowledge is defined using the same ontology, and they aim to align the names they give to the same environment sensors. In Rovatsos et al. (2003), the agents already have a shared negotiation language to communicate, allowing them to negotiate about future actions and behave accordingly.

#### 4.3.2 Interaction components

##### 4.3.2.1 I1. Interaction task

The agent interactions are defined according to some task the agents aim to perform within the interaction. In Kondylidis et al. (2023); Atencia and Schorlemmer (2012), the agents aim to collectively answer a query, where one agent understands the question but the other agent holds the information required to answer it. In Steels and Loetzsch (2012), the agents play the referential game, where one agent describes one of the objects in their environment and the other agent needs to find it by correctly interpreting the communicated description. In Mohan and Laird (2014), the human asks the robot to execute a task physically on the environment. In Euzenat (2014), one agent describes the single object in their shared environment using concepts from its own schema, while the other agent needs to predict the description. In Laera et al. (2007), the agents engage in formal negotiation procedures that help them establish or refine concept alignments when successful. In Anslow and Rovatsos (2015), the agents broadcast observed sensor values to other agents. Lastly in Rovatsos et al. (2003), the agents again follow formal negotiation procedures to negotiate regarding their future actions in a non-committing way.

##### 4.3.2.2 I2. Interaction scenario

The interaction scenario is the situation with which the agents are presented in order to perform their task at the beginning of an interaction. The initiator can be asked to react to a given interaction scenario, as in Steels and Loetzsch (2012); Euzenat (2014); Anslow and Rovatsos (2015), or can design it itself in case of **open** interaction scenario, as in Kondylidis et al. (2023); Mohan and Laird (2014); Laera et al. (2007); Rovatsos et al. (2003). In Steels and Loetzsch (2012), both agents are presented with a random set of objects, and the initiator, i.e. speaker, is informed of the target object that is randomly selected. In Euzenat (2014), the agents are randomly presented with a common instance from their ontologies. In Anslow and Rovatsos (2015), the agent is broadcasting information only when close to a sensor, as the result of its random walk. In Kondylidis et al. (2023); Mohan and Laird (2014); Laera et al. (2007); Atencia and Schorlemmer (2012); Rovatsos et al. (2003), the agents do not have some specific interaction scenario to interact over, and the initiator's communication action is the sole effect of the direction of the interaction.

##### 4.3.2.3 I3. Initiator's acts

The initiator's act describes the action of the agent that acts first within the interaction. This agent is sometimes referred to as “speaker” (Steels and Loetzsch, 2012), “teacher” (Kondylidis et al., 2023), or may not have a particular role. These actions consist of messages that are composed of signals. In Kondylidis et al. (2023), the teacher is communicating two instances, the order of which defines which is a relevant or non-relevant result for the query it tries to explain. In Steels and Loetzsch (2012), the speaker utters a word that is related to a characteristic of one of the objects presented to both agents. In Mohan and Laird (2014), the human tutor is asking the robot to perform a task in natural language. In Euzenat (2014), the agent that initiates the interaction is asking the other agent to describe the randomly selected object using terms of the latter's ontology. (Since this action is always the same, we could have omitted it and assume the agent that replies is the initiator, but we stick to the way the interaction is presented in the original paper). In Laera et al. (2007), the initiator selects the concepts related to the service it wants to perform, focusing on one concept alignment from the alignments' repository that involves any of them, and puts forward arguments in favor or against that alignment. In Atencia and Schorlemmer (2012), the initiator is performing a randomly chosen query defined partly by common ontology concepts and private ones. In Anslow and Rovatsos (2015), the observed sensor value is represented under an ontology in a graph format, and broadcasted as such. In Rovatsos et al. (2003), the initiator begins the negotiation with a request toward the other agent.

##### 4.3.2.4 I4. Respondee's acts

In Kondylidis et al. (2023); Atencia and Schorlemmer (2012), the respondee returns the results of the query, as it interprets it at that time. Additionally, in Kondylidis et al. (2023), the respondee may inform the initiator for the case where the provided example is unclear due to subjective interpretation and cannot be utilized. In Steels and Loetzsch (2012), the respondee replies by selecting an object using the posture of its body. In Mohan and Laird (2014), the robot either performs the next action that it expects to bring the environment closer to the goal state, or asks the human a clarification question about the next action or how the goal state looks like. In Euzenat (2014), the respondee replies with its most “specific” property description of the presented instance, according to its own ontology hierarchy. In Laera et al. (2007), the respondee can also add arguments in favor or against the alignment that is being negotiated. Regarding (Anslow and Rovatsos, 2015), the respondee only reacts in case of uncertainty on interpreting the communicated sensor identity. In that case, the respondee asks the initiator to provide further contextual information about the identity of the communicated sensor. In Rovatsos et al. (2003), the agents engage in a multi-step negotiation based on the original request.

##### 4.3.2.5 I5. Concluding act

In Kondylidis et al. (2023), the initiator is informed by the “environment” how well its query was understood by the respondee. In Steels and Loetzsch (2012), the initiator informs the respondee of the identity of the target object. In Mohan and Laird (2014), the robot can either deduce that it has successfully executed the task, or waits for the human to indicate so. In Euzenat (2014), the agents always communicate in two steps, making the respondee's act to also be the concluding act. In Laera et al. (2007), either agent eventually runs out of arguments and withdraws from the negotiation. In Atencia and Schorlemmer (2012), either agent can terminate the conversation if their conversation interpretation leads them to a terminal state. In Anslow and Rovatsos (2015), in case the initiator is asked to provide further information about the identity of the communicated sensor, i.e., explain the identity, it communicates a graph with further contextual information. In Rovatsos et al. (2003), there is a predefined length of conversation that the agents oblige to.

##### 4.3.2.6 I6. Task performance

One must define how the interaction completion reflects the success of the task performance, since the interaction was performed for a specific task. Then, one can indirectly evaluate how successful the interaction was, and subsequently estimate how well the agents understood each other to the extent that the task required them to. In Kondylidis et al. (2023), the performance of the interaction is indirectly reflected in the query performance using information retrieval metrics. In Steels and Loetzsch (2012); Euzenat (2014); Atencia and Schorlemmer (2012), the outcome of the interaction is either successful or not, resulting in a boolean accuracy. Specifically, in Steels and Loetzsch (2012) the agents communicated successfully if the respondee selected the target object. In Euzenat (2014), the interaction is successful, if the Initiator was able to predict the description that the Respondee would use for this object. In Atencia and Schorlemmer (2012), the interaction is successful if both agents assume that the conversation has ended at the same time, i.e., both reach a finite state in their automata simultaneously. In Mohan and Laird (2014); Laera et al. (2007); Anslow and Rovatsos (2015), there is no evaluation around individual interactions regarding task execution, concept alignment negotiations, or instance matching, respectively. In Mohan and Laird (2014), there is no task performance, since the task is always performed, (with human guidance or not). In Rovatsos et al. (2003), the agents aim to maximize predictability of other agents and as to further maximize their own personal reward. It is important to note that this study does not focus on ensuring shared task-oriented understanding among the agents, but on maximizing the reward of each agent.

#### 4.3.3 Agent policy components

The components that fall under the Agent Policy category concern the agent's behavior and understanding, while also the process of updating them over time.

##### 4.3.3.1 A1. Goal-aware

In Kondylidis et al. (2023); Laera et al. (2007); Rovatsos et al. (2003), the initiator agent is intelligently selecting their Initiator's act, so that they can achieve their goal within a low number of total interactions. In contrast, in Steels and Loetzsch (2012); Mohan and Laird (2014); Euzenat (2014); Atencia and Schorlemmer (2012); Anslow and Rovatsos (2015), the beginning of the interaction is random, leading eventually toward establishing a shared (task-oriented) understanding, but not efficiently.

##### 4.3.3.2 A2. Understanding update

The understanding update module should reflect the learning outcome that is produced after a completed interaction, which should contribute to the goal achievement. At least one agent needs to learn something after an interaction. In Kondylidis et al. (2023); Mohan and Laird (2014); Euzenat (2014); Anslow and Rovatsos (2015), only one of the agents is learning, based on their own assumptions, or also based on provided interpretations as in Mohan and Laird (2014). When both agents learn something, it can be the case that they learn something “similar enough”, or something different. For example, in Steels and Loetzsch (2012); Laera et al. (2007) the agents will have a similar enough learning outcomes, in terms of similar enough visual characteristics or explicitly updating some common public knowledge. In other cases, the agents independently interpret the interaction outcome and make their own assumptions about what they should learn, as in Atencia and Schorlemmer (2012); Rovatsos et al. (2003).

We will now go in the details of what the agents learn in each study. In Kondylidis et al. (2023), the respondee is best approximating the underlying goal-query, after each interaction. In Steels and Loetzsch (2012), both agents reinforce the connection between the communicated word and the visual characteristics of the target object that is revealed by the initiator. It should be noted that the visual characteristics of the target object are expected to be perceived in a similar enough manner. In Mohan and Laird (2014), the respondee (robot) may learn a new task, unless it was asked to perform a task it already knew. Specifically, it retrieves the complete interaction from its episodic memory and in retrospect (i) updates its action outcome estimation model, and (ii) relates tasks with partially described goal states. In Euzenat (2014), the initiator is refining its used concept alignment, in case of a wrong prediction. In Laera et al. (2007), the result of the negotiation can result in adding or removing an alignment from a public ontology alignment repository. In Atencia and Schorlemmer (2012), each agent is re-evaluating the confidence of the alignments they independently used in this specific interaction, according to whether the interaction was successful or not. In Anslow and Rovatsos (2015), only the respondee may update its instance alignments if it manages to successfully induce the target sensor correspondence from the provided explanation. In Rovatsos et al. (2003), the agents are independently updating the expected behavior of the other agent conditioned on the negotiated commitments and the actual performed actions that followed.

##### 4.3.3.3 A3. Understanding evaluation

While the agents interact, there must be a way to scientifically measure the extent to which the agents have established a shared, (task-oriented) understanding. This measurement sometimes depends on how well the agents can perform the task for which they interact, as in Kondylidis et al. (2023); Steels and Loetzsch (2012); Mohan and Laird (2014); Euzenat (2014); Atencia and Schorlemmer (2012). Differently or complementary, some studies evaluate the agent's learned interpretations of the agents, by comparing them with ground truth interpretations, like in Euzenat (2014); Laera et al. (2007); Anslow and Rovatsos (2015). Another way to separate the analyzed studies is depending on whether the agents can alone evaluate their shared (task-oriented) understanding, as for example in Steels and Loetzsch (2012); Mohan and Laird (2014); Euzenat (2014); Atencia and Schorlemmer (2012); Rovatsos et al. (2003). Alternatively, the agents might need external knowledge or input from an oracle, as in Kondylidis et al. (2023); Euzenat (2014); Laera et al. (2007); Anslow and Rovatsos (2015). Euzenat (2014) appears in both categories, as that study uses a combination of evaluations. In Rovatsos et al. (2003), the agents assume to understand other agents according to how certain they are on predicting their behaviors, i.e., minimizing entropy of other agent's action expectations. The setting is competitive and neither the agent reward nor the task require or reflects levels of shared understanding, but the agents can only achieve task-oriented understanding, individually, as they treat the other agent to be part of the environment.

We will now review how the agents evaluate their understanding in each study. Specifically, Kondylidis et al. (2023) uses the performance of the query results to reflect the agents' shared understanding, which in turn is evaluated by an “environment” that acts as an oracle. In Steels and Loetzsch (2012); Atencia and Schorlemmer (2012), the success of the interactions are aggregated over time, turning the task performance into an indirect way to measure shared understanding. In Mohan and Laird (2014), the shared (task-oriented) understanding is measured on how autonomously the agent can perform the task. Since each hint from the human counts as one interaction, levels of shared (task-oriented) understanding are reflected by the inverse of the number of interactions needed to perform the task, aggregated over multiple tasks. In Euzenat (2014), three measures are used. First, the agent should be able to predict the description of the other agent, (task performance). Second, the produced alignments are compared to ground truths and alignments produced by other methods (external validation). Third, they measure the level of inconsistency in the produced alignments (as an introspective evaluation). In Laera et al. (2007); Anslow and Rovatsos (2015), the agent's produced alignments are compared to ground truths.

##### 4.3.3.4 A4. Efficiency evaluation

The task performance reflecting the levels of achieved shared understanding may be the main focus, but one must evaluate that with respect to the cost of the method. Unfortunately, most of the analyzed studies overlook this aspect. Specifically, some studies do not measure nor make any report regarding the cost of their method (Laera et al., 2007; Rovatsos et al., 2003). Other studies report how their method behaves with respect to the number of interactions, only to show how the experiment behaves over time (Steels and Loetzsch, 2012; Euzenat, 2014). The remaining studies do consider the method's cost as an important factor and use it as an additional way to compare the presented methods. Kondylidis et al. (2023) reports the method performance with respect to both the number of interactions and the memory demands of the method as an example of measuring the cognitive load of an agent. In Mohan and Laird (2014), the proposed method aims to balance between generalizing well, i.e., requiring less interactions to learn new tasks, and its own computational effort used for search space exploration, measured in decision cycles. Atencia and Schorlemmer (2012) and Anslow and Rovatsos (2015) compare the proposed methods' performance with respect to the number of interactions and the amount of total communicated volume, respectively.

### 4.4 Establishing shared understanding analysis

Here we answer our second research question, “how can our framework definitions help us understand what kind of understanding the agents are establishing.” This is an outcome of the earlier analysis of the studies per component of our framework. Specifically, P4. Prior Understanding allows us to clarify what understanding did the agents shared prior to interacting. Analysis of I1. Interaction Task allow us to decide whether the task requires collaboration and whether shared understanding is reflected on I6. Task Performance. A2. Understanding Update analysis helps us identify whether the agents' understanding is expected to be more similar after an interaction. This way, we can describe what types of (task-oriented) understanding are provided to the agents and what types are established throughout the interactions. A summary of our findings is illustrated at the bottom of Table 2, although we go over each row separately in the following paragraphs.

#### 4.4.1 Individual task-oriented understanding

In Steels and Loetzsch (2012), the agents need to develop internal representations that allow them to discriminate objects that are presented to them, therefore the task-oriented understanding of the individual agents is **established** throughout the interactions. Regarding Mohan and Laird (2014), we focus on the agent that learns, i.e., the robot, who does not know how to perform the tasks alone, and therefore its individual task-oriented understanding is **established**. In Rovatsos et al. (2003), the agent needs to be able to estimate the behavior of other agents in order to receive high personal reward. In this case, other agents are treated as part of the environment and the agent needs to understand their behavior for its own gain. We consider this as an **establishment** of individual task-oriented understanding. On the other hand, in Kondylidis et al. (2023); Euzenat (2014); Laera et al. (2007); Atencia and Schorlemmer (2012); Anslow and Rovatsos (2015) the agents are already **provided** with a static way to perceive their environment, i.e., ontology concepts, and do not further refine their individual task-oriented understanding.

#### 4.4.2 Shared task-oriented understanding

In most of the presented studies, the agents **establish** shared task-oriented understanding through interaction, as in Kondylidis et al. (2023); Steels and Loetzsch (2012); Mohan and Laird (2014); Euzenat (2014); Atencia and Schorlemmer (2012); Anslow and Rovatsos (2015). This is **not the case** for Laera et al. (2007); Rovatsos et al. (2003). In Laera et al. (2007), the agents are provided with a formal negotiation framework in order to negotiate regarding term alignments, since that is what I1. Interaction Task requires them. Therefore, the agents do not establish or extend their understanding in order to perform the I1. Interaction Task, but instead it is already provided. In Rovatsos et al. (2003), the I1. Interaction task, does not require agent cooperation and therefore by definition, the agents cannot establish shared task-oriented understanding. Instead, the agents only refine their individual task-oriented understanding, but there is no way to know if they do not establish shared task-oriented understanding.

#### 4.4.3 Shared understanding

In all the presented studies except for Rovatsos et al. (2003), the agents develop shared understanding. In Kondylidis et al. (2023); Euzenat (2014); Laera et al. (2007); Atencia and Schorlemmer (2012); Anslow and Rovatsos (2015), the agents learn to represent ontology concepts in similar enough manner. In Steels and Loetzsch (2012), the agents develop representations of similar enough visual features that they relate with the same words. In Mohan and Laird (2014), the human is satisfied with how the robot is executing the tasks in terms of outcomes observed on the physical environment. This allows us to assume that their task understanding, both in terms of subtasks involved and acceptable goal states, is similar enough. In Rovatsos et al. (2003), it is possible that the agents develop similar internal representation regarding agent action commitments and following actions. However, whether this is the case remains **undetermined**, since we cannot measure levels of shared understanding directly, i.e., comparing internal representation nor indirectly. Levels of shared understanding can be indirectly measured using I6. Task Performance, only when the latter requires agent cooperation, which is not the case for the competitive setting presented in Rovatsos et al. (2003). The agents do attempt to increase the certainty of predicting other agents' actions based on their uttered commitments, which can be interpreted as establishing shared understanding, but their main objective is still to maximize their own reward.

### 4.5 Hybrid applicability of presented studies

Here, we answer our third research question, i.e., whether our framework definitions can help us evaluate the applicability of an OMAS shared understanding establishment study to a hybrid population. Continuing our case-by-case analysis, we will now analyze which studies are compatible to be applied in hybrid populations, or point out their limitations and suggest solutions in case they are not. The summary of our findings is presented in the last row of Table 2.

#### 4.5.1 Restriction 1: physical and concise communication

We assume that the digital communication signals (P3. Signals) can be trivially translated to have a physical form, e.g., presented as symbols or words on a monitor, so we do not see this as a restriction for any of the studies. On the other hand, communication acts (I3–I5), are not always concise in the analyzed studies. In Kondylidis et al. (2023); Steels and Loetzsch (2012); Euzenat (2014); Atencia and Schorlemmer (2012), the agents communicate using a handful of symbols, single words or body postures to point toward some object, making their communication acts concise so that they can also be used when interacting with humans. In Mohan and Laird (2014), the agents communicate using natural language over a chat interface, making it a good example of concise and physical communication. In Laera et al. (2007), the communicated symbol correspondences are small in terms of number of symbols used, but may be hard to interpret and assess by a human in case the ontology is large. A way to resolve this would be to reduce the complexity of the communicated arguments, or the number of possible arguments per negotiated correspondence, although this could affect the performance of the presented application. In Anslow and Rovatsos (2015), the optional explicatory request is responded to with an explanation, where the information is represented in graph form. This might not be very intuitive in terms of interpretation effort, and may not be very human-compatible. This could be tackled by putting restrictions on the size of the communicated graph, although the authors aim to reduce it already. Additionally, the agents could communicate a map visually depicting their current local position with the observed sensor values indicated on it. Furthermore, the authors point out that their method could not be directly applied with human participants, unless a common ontology is provided to all agent participants. In Rovatsos et al. (2003), the agents follow simple negotiation protocols that do not exceed a total of three negotiating steps. The messages therefore are concise enough to be used by humans, although it is not clear if a human would be able to learn from such interactions, due to the large number of possible action interpretations. This can be overcome by limiting the application space to simple interaction games with few possible actions and a small and discrete space of returns.

#### 4.5.2 Restriction 2: Task Performance Reflects Shared Understanding

Since the interpretations of a human cannot be directly accessed and evaluated, the Task performance must reflect the levels of shared (task-oriented) understanding between the agents. In Kondylidis et al. (2023); Steels and Loetzsch (2012); Mohan and Laird (2014); Euzenat (2014); Atencia and Schorlemmer (2012), the task performance can be used to indirectly reflect levels of shared (task-oriented) understanding, allowing their application in hybrid populations. In Laera et al. (2007), the I1. Interaction Task, is to complete an ontology term alignment negotiation and does not reflect the correctness of either the negotiation outcome or of the complete set of accepted alignments. For this reason, the produced alignments are externally validated by comparing them to a provided set of ground truth correct alignments. This would **not** be applicable for a hybrid population, as the ground truth alignments could not be put together or used for comparison and evaluation. This can be overcome by measuring the success of the original service that requires the agents to align their ontologies, and use that to indirectly estimate levels of shared understanding; turning the ontology alignment task to a task-oriented ontology alignment task. The case is similar for Anslow and Rovatsos (2015), where the task is not evaluated and the produced instance alignments are validated externally. Once more, the agents could make the needed instance alignment to be a requirement for some downstream task, allowing it evaluation to reflect levels of correct instance alignments. As mentioned in Section 4.4, the I1. Interaction Task used in Rovatsos et al. (2003) does not require the agents to cooperate. Therefore, the task performance does not reflect levels of shared (task-oriented) understanding regardless of whether it is applied to a hybrid population or not. In order for the task to reflect levels of shared understanding in this case, the agents need to engage in a game of pure common interest.

#### 4.5.3 Restriction 3: efficiency is evaluated

Only Kondylidis et al. (2023); Mohan and Laird (2014); Anslow and Rovatsos (2015); Atencia and Schorlemmer (2012) point out that the cost of establishing shared (task-oriented) understanding is important and measure it. Kondylidis et al. (2023); Atencia and Schorlemmer (2012) measure the number of interactions needed. Kondylidis et al. (2023) also presents an example of how to measure the cognitive effort needed by the agent participants to understand each other. Mohan and Laird (2014) additionally highlights the trade-off between minimizing the number of interactions and reducing the computational load of the robotic agent, enabling adaptation to specific use cases. Anslow and Rovatsos (2015) measures the conciseness of the communicated messages in terms of communication volume. The rest of the presented studies do not emphasize or report the cost of establishing shared (task-oriented) understanding, and would have to adapt by for example counting the number of interactions, or estimating the cognitive load of the participants in order to ensure their efficient application in hybrid populations.

## 5 Conclusions, discussion, and future work

In this study, we first put forward the definitions of individual task-oriented understanding, shared task-oriented understanding and shared understanding in Section 3.2. We then provide a framework describing the process through which two agents establish shared (task-oriented) understanding in Section 3.3, answering our first research question: *Can we represent the process of establishing shared understanding in a framework?*. Next, in Section 4.3, we use our framework to analyze existing OMAS studies from diverse domains, exhibiting its generalisability and allowing us to later decide in which of the studies the agents establish shared (task-oriented) understanding in Section 4.4, answering our second research question: *Can this framework help us identify whether two agents indeed establish shared understanding?* Finally in Section 4.5, we use our earlier framework application to detect limitations of the analyzed studies prohibiting their application in hybrid populations, answering our third research question: *Can this framework allow us to foresee limitations of existing studies if they were to be applied in hybrid populations?*

While the proposed framework describes interactions between two participants, in principle it can also be applied to larger agent populations, as long as they interact in pairs. This is also exhibited in language games for example Steels and Loetzsch (2012), where eventually every pair of the population can successfully play the referential game, while they only interacted in pairs at all times and ignoring the identity of the agent they interact with. Nevertheless, the higher the heterogeneity of the population, in terms of environment perception due to for example using different sensors, or focusing on performing different tasks, establishing shared (task-oriented) understanding is expected to be increasingly challenging. This can be perceived as a complex dynamic system needing more time to converge to a semantic equilibrium. This process can be additionally challenging if the agents interact in clusters, i.e., have higher probability to interact with some part of the population compared to the rest. Similarly, in dynamic agent populations, agents re-entering after a long absence should not assume that previously established shared (task-oriented) understanding still holds, as the population's interpretations may have shifted in the meantime.

We suggest that future directions of this research can include the following. First, research should focus on enriching ways to measure shared understanding. Our proposed shared task-oriented understanding, which is a tool to indirect measure shared understanding, can only be measured if the agents engage in a game of pure common interest. Different definitions or tools to measure shared understanding should be proposed for measuring shared understanding in different agent interaction settings, like in competitive ones. An alternative could be to compare the agents' behavior, if they were put in the same situation. Second, our A4. Efficiency Evaluation can be extended with more specific guidelines on estimating the cognitive load of participating human agents and determining acceptable levels, based on related literature. Finally, in an OMAS, agents can interact with a number of other agents and for different goals over time. In such case, the application of the presented framework, will lead them to establishing shared (task-oriented) understandings for each agent-goal combination separately and from scratch. In order to minimize this overhead, our framework can include some forms of theory of mind representations (Colombo and Piccinini, 2023), allowing the agents to speed up the process of establishing shared task-oriented understanding for a new agent-goal combinations, based on prior experience with similar agents or goals. For example, an agent can choose to focus on aligning more abstract or fine-grained ontology concepts, depending on the agent they interact with, e.g., whether it is an expert mechanic or some end-user, or which vocabulary to use depending on the group of people the other agent belongs to.

## Data Availability

The original contributions presented in the study are included in the article/supplementary material, further inquiries can be directed to the corresponding author.
